# Study on Effects of Refining Slag on Properties and Hydration of Cemented Solid Waste-Based Backfill

**DOI:** 10.3390/ma15238338

**Published:** 2022-11-23

**Authors:** Chang Tang, Xinli Mu, Wen Ni, Dong Xu, Keqing Li

**Affiliations:** 1 School of Civil and Resource Engineering, University of Science and Technology Beijing, Beijing 100083, China; 2 Key Laboratory of the Ministry of Education of China for High-Efficient Mining and Safety of Metal Mines, University of Science and Technology Beijing, Beijing 100083, China; 3 Beijing Key Laboratory on Resource-Oriented Treatment of Industrial Pollutants, University of Science and Technology Beijing, Beijing 100083, China; 4 Nanjing Institute of Environmental Sciences, Ministry of Ecology and Environment, Nanjing 210042, China

**Keywords:** refining slag, solid-waste cementitious material, cemented backfill, hydration kinetics

## Abstract

This study used refining slag (RS), ground granulated blast furnace slag (GGBS), steel slag (SS), and desulfurized gypsum (DG) to prepare a mine-filling cementitious material. The developed cementitious material and tailings sand were mixed to prepare a novel mine backfill material with better performance and a lower cost. The macroscopic properties and hydration mechanism of the cemented solid waste-based backfill were investigated when RS content was 0, 5%, 10%, 15%, 20%, 30% and 40%. The results showed that introducing RS could reduce the bleeding rate and shorten the setting time of backfill slurry while significantly enhancing the 3-day compressive strength of backfill. Compared to JL-0, the bleeding rate decreased by 50.3% as the RS content was raised to 15%, while the setting time was shortened by 36.5%, and the 3-day compressive strength increased by 4.3 times. As the RS content did not exceed 20%, the 28-day compressive strength of the backfill was not lower than that of the cement backfill (4.3 MPa). The results of microanalysis (including XRD, FT-IR, SEM, TG-DSC, and heat of hydration) revealed that the hydration products of the RS-GGBS-SS-DG quaternary material are primarily C-(A)-S-H gels and AFt. The main effect of RS is to improve the content of aluminates, accelerating and increasing the production of AFt, thus leading to faster overall hydration. This research can provide data support for the application of RS in the mine-filling field. Applying quaternary solid waste-based cementitious materials in the mine-filling field has good economic benefits.

## 1. Introduction

Owing to its high safety, low ore loss, and flexible engineering operation, the filling mining method has gradually become one of the main mining technologies. However, the central issues hampering its applicability are the high cost of backfill materials (with cement as the primary binding material), accounting for about 1/3–1/2 of the mining cost [1], and the large amount of solid waste (ore and tailings sand) from mines, which causes great ecological and environmental pollution to the surrounding water and soil [2]. For a long time, finding cheaper cement-substitutes to prepare mining-filling cementitious materials and exploring how to consume a large amount of mine waste have been hot research topics.

Using industrial solid waste as cement binder replacement to prepare mine backfill materials is an effective way to reduce filling costs and attain sustainable development in mining [3]. In this regard, a variety of industrial solid wastes have been used for the preparation of mine-filling cementitious materials, such as ground granulated blast furnace slag (GGBS) [4], steel slag (SS) [5], fly ash [6], and red mud [7]. Li et al. [8] utilized oil shale residue (OSR), SS, and GGBS to create an environmentally-friendly cementitious material for mine-filling. The optimal mix proportions were obtained by the response surface method, and the resulting 28-day compressive strength of backfill material can reach up to 2.12 MPa for 4.85% GGBS content, SS: OSR = 0.82, and 67.69% slurry. Ibrahim et al. [9] used 25% ordinary Portland cement (OPC) and 75% fly ash as the cementitious material with sodium silicate as the activator, and the resulting 28-day compressive strength of the developed mine-filling material reached more than 10 MPa.

In the studies on binders developed from industrial solid wastes, a ternary cementitious material consisting of GGBS, SS, and DG has been widely investigated and applied to concrete and mine filling. Xu et al. [10] studied the hydration reaction of GGBS-SS-DG ternary cementitious material and found that the hydration products of this system were mainly C-S-H gels and ettringite (AFt). Zhang et al. [5] prepared a binder consisting of 50% GGBS, 35% SS, and 15% DG. With the binder/tailings ratio of 1:4 and slurry concentration of 78%, the 3-day and 28-day compressive strengths of the backfill were 6.69 MPa and 16.36 MPa, respectively. However, some researchers have shown that the GGBS-SS-DG ternary cementitious material has a slow hydration rate at the early stages, resulting in a low compressive strength [11]. Li et al. [12] used refining slag (RS) instead of SS to prepare the GGBS-RS-DG ternary cementitious material and found that the system has a more rapid early-stage reaction and better early-age compressive strength. Liu et al. [13] further introduced RS into the GGBS-SS-DG ternary cementitious material to prepare a quaternary cementitious system, demonstrating that RS can effectively accelerate the early-age hydration reaction of cementitious materials and enhance the 3-day compressive strength of concrete test blocks. However, the research findings on the preparation of backfill materials with this quaternary cementitious material have not been reported yet.

Refining slag (RS) refers to the waste slag discharged in further refining crude steel to prepare refined steel. Unlike SS, RS contains a large amount of Mayenite (12CaO·7Al_2_O_3_, C_12_A_7_) [14,15]. Mayenite has a fast reaction speed in the liquid phase and quickly generates ettringite (AFt phase), especially in the presence of SO_4_^2−^ and Ca^2+^ [16,17,18,19,20,21]. Although RS has been utilized to prepare cement and concrete, its current utilization rate is not high because of its rapid hydration reaction and some harmful components that limit the amount added to building materials [12,22]. In order to attain an increased early-age hydration rate of GGBS-SS-DG ternary cementitious material, consume/utilize RS waste, and reduce the economic cost of backfill material, it is imperative to develop the RS-GGBS-SS-DG quaternary cementitious material for mine filling.

In order to solve the problems in the practical application of GGBS-SS-DG ternary cementitious solid waste-based material, this study developed and optimized the RS-GGBS-SS-DG quaternary cementitious material utilizing theoretical research on the ternary cementitious material system. Furthermore, a new environmentally-friendly backfill material was prepared using RS-GGBS-SS-DG quaternary cementitious material to cement tailings sand. The effects of RS addition on the performance and mechanical properties of the cemented backfill were studied. The study on the microstructure and hydration reaction mechanism of solid waste-based cementitious materials is of great significance for revealing the reasons for the change of working properties of backfill materials and guiding the practice of backfill engineering. The hydration hardening and strength growth mechanism of the solid waste-based quaternary cementitious material was analyzed by XRD, FT-IR, TG-DSC, SEM, heat of hydration analysis, and other characterization methods. The effects of RS content on the hydration and properties of cementitious material are thoroughly discussed. Studying the properties and hydration mechanism will provide a theoretical foundation for the engineering application of this material preparation technology. Based on the macro-strategic requirements of mining and metallurgy solid waste recycling and carbon emission reduction, and the realistic demand of backfill material cost of mining-filling technology, the objective of this paper is to corroborate the use of refining slag and other solid wastes to prepare environmentally-friendly mine filling materials with better performance, better environmental protection benefits, and lower cost, providing a basis for the RS application in backfilling.

## 2. Materials and Methods

### 2.1. Raw Materials

#### 2.1.1. Cementitious Material

**Figure 1:**
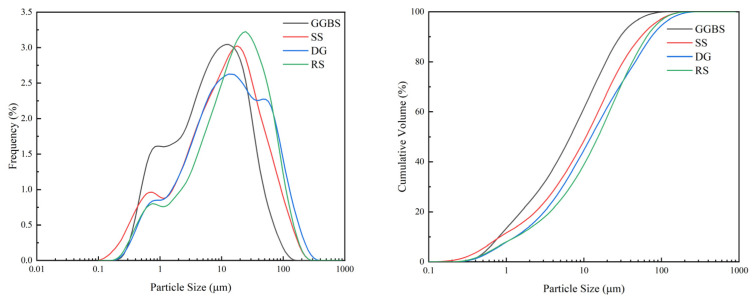
Particle size distribution of raw materials.

**Figure 2:**
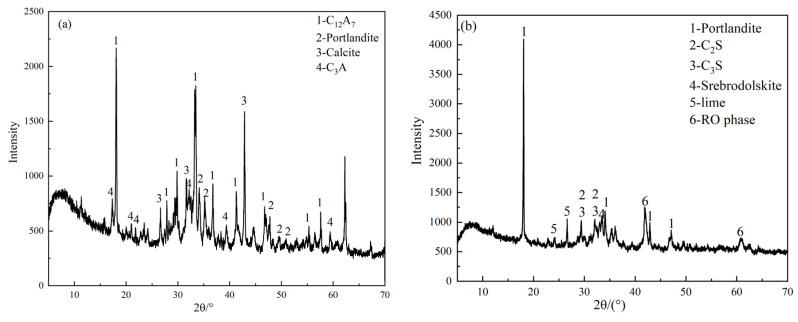

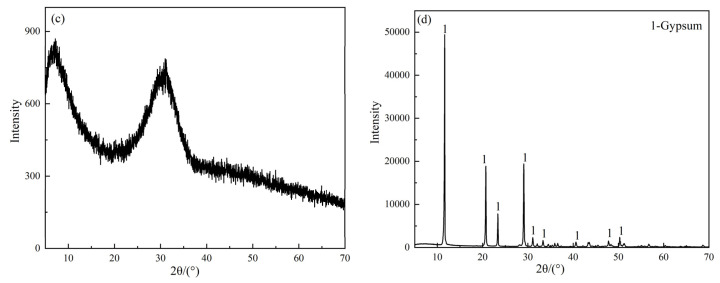
XRD patterns of raw materials: (**a**) RS; (**b**) SS; (**c**) GGBS; (**d**) DG.

The cementitious materials used in the test were prepared from RS, SS, GGBS, and DG, with specific surface areas of 415 m^2^/kg, 480 m^2^/kg, 515 m^2^/kg, and 420 m^2^/kg, respectively. The particle size distribution is shown in Figure 1. The four raw materials were obtained from Han-Steel Group, Delong Steel Group, and Hebei Shahe Power Plant. Table 1 shows their chemical compositions, and Figure 2 depicts their XRD patterns. In addition, OPC was used as the control group.

RS refers to the waste slag generated during the production of refined steel in an LF ladle furnace, and has a higher alumina (Al_2_O_3_) content of 18.20%. XRD analysis (Figure 2b) shows that the mineral phases of RS are primarily aluminates, including Maynite (C_12_A_7_) and tricalcium aluminate (C_3_A).

SS refers to the steel slag discharged during converter steel making. Its main mineral phases are calcium hydroxide (Ca(OH)_2_), dicalcium silicate (C_2_S), tricalcium silicate (C_3_S), and RO phase. Its alkalinity M was calculated as 2.15 from Equation (1) [23], indicating that it is a high-alkalinity steel slag with higher activity.

From Equations (2) and (3), the alkalinity coefficient M_O_ of GGBS was found to be 1.11, so it belonged to alkaline slag. The quality coefficient K was 2.02, which belonged to highly active slag. The XRD pattern of GGBS is shown in Figure 2c, with no obvious characteristic peaks, indicating that its main mineral phase is amorphous and glassy.

The main chemical components of DG are CaO and SO_3_, and the main mineral phase is identified as gypsum (CaSO_4_·2H_2_O).

(1) $$M=\frac{w_{\mathrm{CaO}}}{{(w}_{{\mathrm{SiO}}_{2}}{+w}_{P_{2}O_{5}})}$$

(2) $$\mathrm{Mo}=\frac{{(w}_{\mathrm{CaO}}{+w}_{\mathrm{MgO}})}{{(w}_{{\mathrm{SiO}}_{2}}{+w}_{{\mathrm{Al}}_{2}O_{3}})}$$

(3) $$K=\frac{{(w}_{\mathrm{CaO}}{+w}_{\mathrm{MgO}}{+w}_{{\mathrm{Al}}_{2}O_{3}})}{{(w}_{{\mathrm{SiO}}_{2}}{+w}_{{\mathrm{TiO}}_{2}}{+w}_{\mathrm{MnO}})}$$

where w denotes the mass fraction of each chemical component.

#### 2.1.2. Tailings Sand

**Figure 3:**
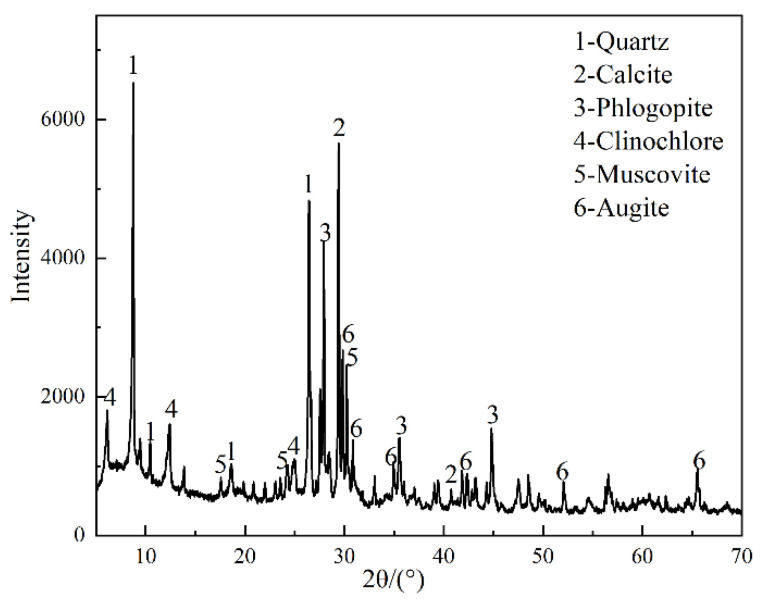
XRD pattern of tailings sand.

**Figure 4:**
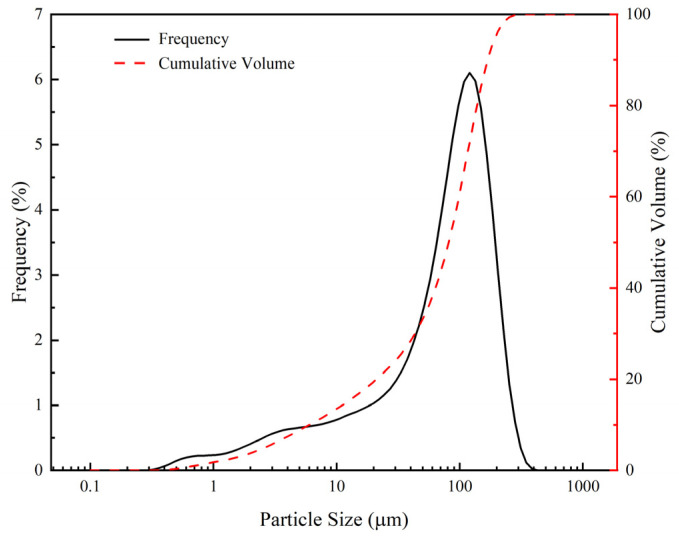
Particle size distribution of tailings.

The tailings sand used in the study was obtained from HBIS Group. According to Table 2, the main chemical components were CaO and SiO_2_. Quartz and calcite mineral phases were identified from the XRD analysis (Figure 3). The particle size distribution of the tailings sand is shown in Figure 4.

### 2.2. Specimens Preparation

RS, SS, GGBS, and DG were used as cementitious materials, whereas the tailings sand was used as fine aggregate to prepare cemented solid waste-based backfill. The performance variation of the backfill materials as well as the hydration mechanism and strength development of the solid waste-based quaternary cementitious material system, was studied when RS replaced different proportions of GGBS. Table 3 shows the mix proportion. P.O.42.5 cement with an equal amount added was used as the OPC sample and the auxiliary reference for comparison.

Preparation of backfill specimens: The cement-sand ratio of the backfill material was kept at 1:3, and the slurry concentration was 70%. The cementitious materials, tailings, and water were weighed in the specified proportions and evenly mixed to obtain a filling slurry. The slurry was cast into the 70.7 × 70.7 × 70.7 mm molds. The specimens were demolded after 24 h to obtain the test block of backfill. After 3, 7, and 28 days of curing at the standard curing conditions (20 ± 1 °C and RH > 95%), the specimens’ strength was tested.

Preparation of paste specimen: According to the ratios of JL-0–JL-30 shown in Table 3, each component was weighed in proportion. A constant water-to-binder ratio of 0.4 was used. The cementitious materials and water were evenly mixed, cast into the 30 mm × 30 mm × 50 mm molds, and subjected to the standard curing conditions of 20 ± 1 °C and RH > 95%. After 24 h, the specimens were demolded. The paste specimen was removed at the desired testing age and hydration terminated. Then the paste was broken into powder and sheets for microscopic investigation and characterization (including XRD, SEM, TG-DSC, and FT-IR) of the hydration products of cementitious materials.

### 2.3. Methods

#### 2.3.1. Macroscopic Properties Test

The mechanical property testing for the backfill was conducted based on GB/T 50080-2016 [24] and JGJ/T70-2009 [25]. The fluidity test for backfill was carried out according to GB/T 2419-2005 [26], where a slump cone with an upper diameter of 70 mm, a lower diameter of 100 mm, and a height of 60 mm was used. The setting time of backfill was determined according to JGJ/T 70-2009 [25].

The bleeding rate of the backfill slurry was determined as follows: A certain mass of mixed backfill slurry (m_1_) was placed into a beaker for two hours, from which the bleeding water was quickly sucked out with a pipette. The remaining slurry weight (m_2_) was measured. Equation (4) shows the calculation method for the bleeding rate.

(4) $${n=(m}_{1}-m_{2}{)/m}_{0}$$

where $m_{0}$ denotes the total water consumption of the backfill slurry as weighed.

#### 2.3.2. Microscopic Characterization

X-ray fluorescence analysis (XRF) was carried out by XRF-1800 scanning X-ray fluorescence spectrometer (Shimadzu Co., Ltd., Kyoto City, Japan). X-ray diffraction analysis (XRD) was performed using a D/MAX-RC 12KW powder X-ray diffractometer (Rigaku. Japan). The diffractometer adopted a Cu target and used a working voltage of 40 kV, a working current of 150 mA, and a scanning angle range of 2θ = 5°–90°. Fourier Transform Infrared ray diffraction analysis (FT-IR) was conducted by Nexus 70 Fourier infrared spectrometer (Thermo Nicolet Company, Madison, WI, USA), with a scan range of 350 cm^−1^–7000 cm^−1^ and a resolution of 3 cm^−1^. A scanning electron microscope (SEM, Suppatm 55, Carl Zeiss, Oberkochen, Jena, Germany) with an accelerating voltage of 0.02 kV–30 kV was employed to observe the surface morphology. The TAMAir eight-channel micro-calorimeter was used to analyze the hydration heat released by the specimen continuously at 20 °C. The thermogravimetric analysis and differential scanning calorimetry (TG-DSC) measurements were done by a thermogravimetric analyzer (Netzsch STA 449C, Selb, Bavaria, Germany). The temperature range was 20–1000 °C, and the heating rate was 10 °C/min under a nitrogen environment.

## 3. Results and Discussion

### 3.1. Single-Factor Test Results of RS Content

#### 3.1.1. Bleeding Rate of Backfill Slurry

**Figure 5:**
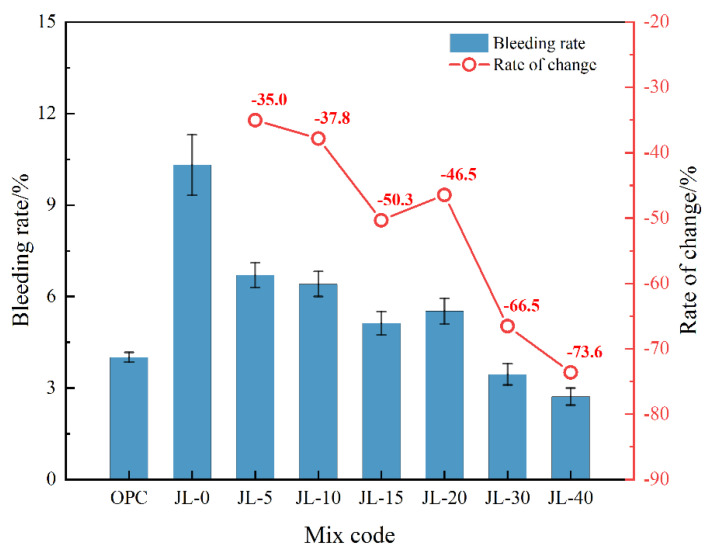
Effect of RS content on bleeding rate of backfill slurry.

Figure 5 depicts the bleeding rate comparison of the cemented solid waste-based backfill slurry between the OPC and JL-0–JL-40 samples. With the increase in RS content, the bleeding rate of backfill slurry shows a downward trend. This can be attributed to the rapid hydration reaction of aluminate minerals (C_12_A_7_ and C_3_A) in the RS, which consumes a large amount of free water. When the RS content increases from 0 to 5%, the bleeding rate decreases by 35%, indicating that adding RS can greatly decrease the bleeding rate of the solid waste-based cementitious system.

Compared to the OPC sample, the JL-0 sample without RS has a higher bleeding rate and is easy to segregate. However, the bleeding rate decreases after adding RS. The bleeding rate of samples incorporating >30% RS is close to that of the OPC sample, indicating a reduction of over 66.5% compared to the JL-0 sample. Thus, it can be inferred that RS addition can improve the bleeding performance of backfill consisting of solid waste-based cementitious material, so that it reaches the same consistent performance level as the cement material.

#### 3.1.2. Fluidity of Backfill Slurry

**Figure 6:**
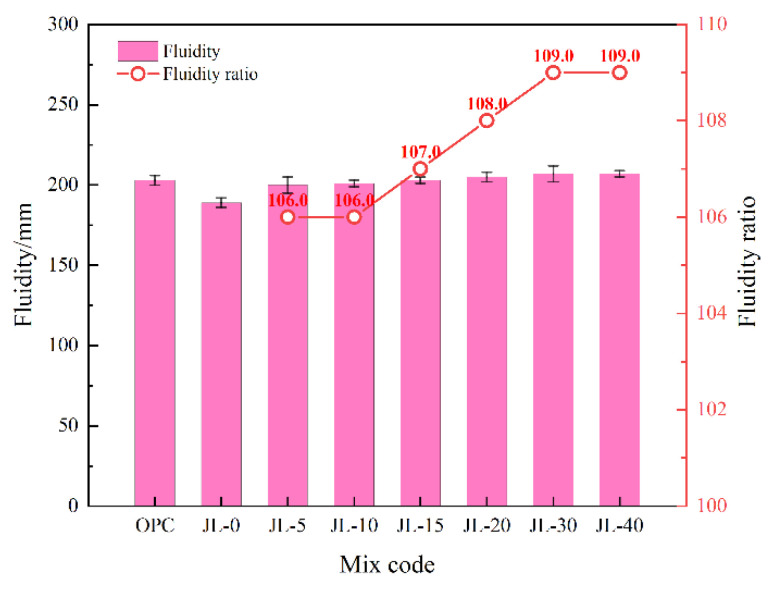
Effect of RS content on the fluidity of backfill slurry.

Figure 6 indicates that the fluidity of the backfill slurry increases slightly with the increase in RS content, indicating that the addition of RS is conducive to improving the fluidity of the backfill slurry to a certain degree. However, the fluidity of JL-40 is almost identical to that of JL-30, showing no enhancement, so when RS content is higher than 30%, the enhancement in fluidity is limited. The phenomenon can occur because when the RS amount exceeds a particular proportion, it is easy to form a flocculent structure in the slurry, thereby reducing the fluidity.

The fluidity of the backfill consisting of ternary cementitious material (JL-0) is 189 mm, while that of the OPC sample is 203 mm, demonstrating a specific difference. Figure 6 illustrates the ratio of the fluidity of each backfill sample to that of the JL-0 sample. It can be seen that the fluidity of the solid waste-based cementitious materials increases after mixing with RS; all samples with RS exhibit higher fluidity than the JL-0 sample and are close to the OPC sample. The fluidity of the JL-15 and OPC samples is the same, and the fluidity of the JL-20–JL-40 samples is higher than that of the OPC sample, indicating that their fluidity performances are better than cement. Although RS can increase the fluidity of backfill slurry, the change range is not large; thus, the influence can be neglected.

#### 3.1.3. Setting Time of Backfill

**Figure 7:**
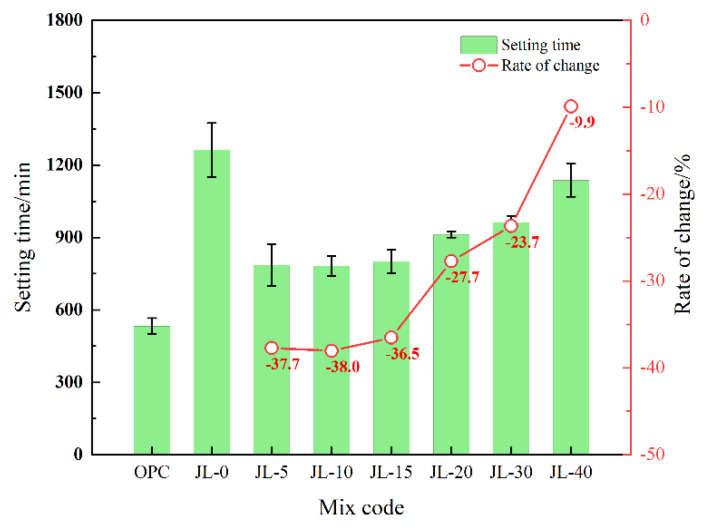
Effect of RS content on the setting time of backfill.

As shown in Figure 7, the setting times of all the solid waste-based cementitious material samples are longer than that of the OPC sample, in which the JL-0 sample has the longest setting time. It shows that the hydration rate of the solid waste-based cementing material is lower than that of cement. Based on the setting time of JL-0, Figure 5 shows the curve of the setting time change rate of JL-5–JL-40 samples. Compared to the JL-0 sample, the setting time of JL-5 decreases by 37.71%, indicating that RS addition helps greatly reduce the setting time of solid waste-based cementitious material. The setting times of JL-5 to JL-15 samples are stable at about 800 min; they are 37.7%, 38.0% and 36.5% lower, respectively than that of JL-0. Based on the observation of JL-20–JL-40 samples, the setting time is gradually extended with the continuous increase in RS content. The setting time of JL-40 is only 9.9% shorter than that of JL-0, but it can still meet the mine’s production requirements (not longer than 24 h). It can be seen that RS has a remarkable effect on shortening the setting time of solid waste-based cementitious material as cemented backfill when the RS content ranges between 5% and 15%. Nevertheless, the improvement is insignificant when RS content exceeds 15%.

#### 3.1.4. Compressive Strength of Backfill

**Figure 8:**
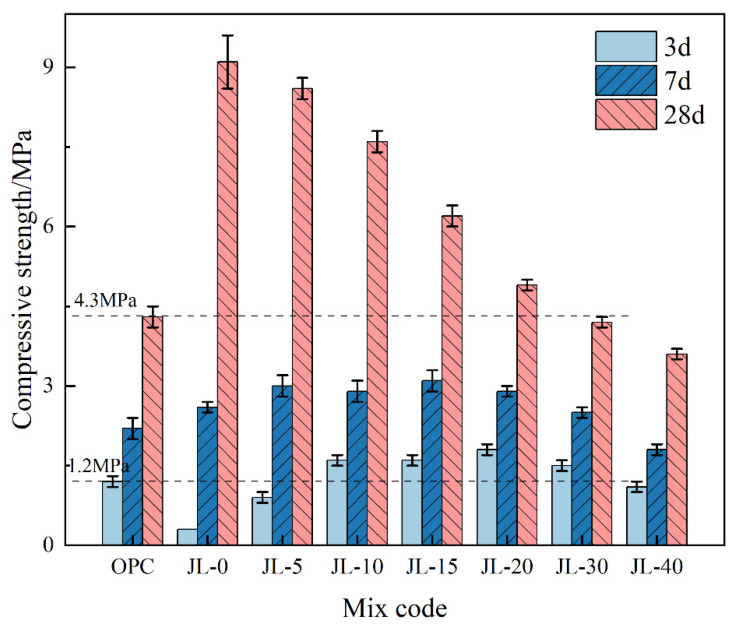
Effect of RS content on the compressive strength of backfill.

Figure 8 shows the 3-day, 7-day, and 28-day compressive strengths of cemented solid waste-based backfill samples and cemented OPC backfill samples. By comparing the 3-day strength of each sample, it can be seen that the compressive strength of the backfill is significantly enhanced by adding RS. The strength of JL-15 is 4.3 times that of JL-0, but the compressive strength is reduced when the RS content exceeds 20%. Compared to the OPC sample, the 3-day strengths of JL-10–JL-30 samples are higher, with an increase of about 20%. It can be seen that the RS addition effectively solves the problem of inadequate early-age strength, which is usually observed in cemented solid waste-based backfill.

The 7-day compressive strengths of JL-0–JL-30 samples are higher than those of OPC, and only that of JL-40 is slightly lower. As the RS amount varies, the variation trend of compressive strength of backfill is consistent with that of 3-day compressive strength. The 28-day compressive strength of JL-0 is twice that of OPC, indicating that compared with cement, the hydration reaction of solid waste-based cementitious materials lasts longer. With the increase in RS content and the decrease in GGBS content, the 28-day strength gradually decreases, but is still higher than that of OPC (4.3 MPa) as the RS content does not exceed 20%. Therefore, RS negatively impacts the strength enhancement at the later stages and cannot contribute the same amount of hydration products as GGBS. Overall, considering that the compressive strengths of cemented backfill at each curing age are higher than OPC, the RS content should preferably range from 10% to 20%.

### 3.2. Micro-Analysis Results of Solid Waste-Based Cementitious Materials with Different RS Contents

#### 3.2.1. XRD Analysis

**Figure 9:**
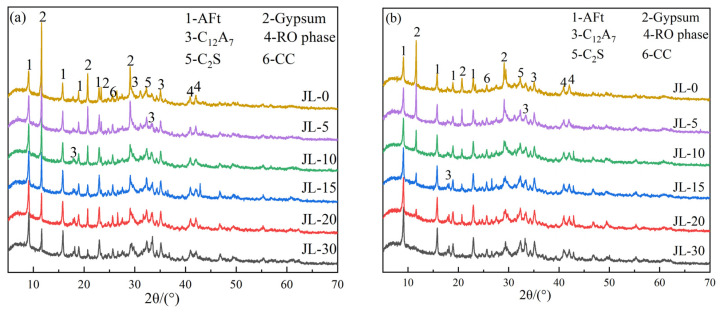
XRD patterns of solid waste-based cementitious material: (**a**) 3 d; (**b**) 28 d.

Figure 9 shows the XRD patterns of solid waste-based cementitious materials with different RS contents curing for 3 and 28 days. It can be seen from the XRD pattern in Figure 2a and Figure 9 that C_12_A_7_ has a higher characteristic peak intensity in RS as raw material, but only a weak peak intensity is presented in the 3-day and 28-day specimens, indicating that C_12_A_7_ is rapidly consumed during hydration. The peak intensity of C_12_A_7_ in the 28-day specimen is slightly lower than in the 3-day specimen, which shows that its hydration mainly occurs in 0–3 days, and the remaining small amount of C_12_A_7_ is wrapped by the products formed during the hydration process, causing a slow reaction.

The characteristic peak intensity of DG decreases with the increase in RS content, indicating that RS promotes gypsum consumption. In addition, the characteristic peaks of AFt can be observed in all specimens, and the intensity increases with increasing RS content, indicating that RS can promote the formation of AFt. It can be seen from the comparison between Figure 9a,b that with the increase in curing age, the peak intensity of AFt increases, and the peak intensity of gypsum decreases. Meanwhile, the JL-30 sample has almost no characteristic peak of gypsum at 28 days. This indicates that the hydration reaction proceeds continuously, consumes gypsum, and generates AFt and other products. In addition, all specimens in Figure 9 have a broad, convex peak at 2θ = 25°–35°, indicating the presence of amorphous C-(A)-S-H gels [27,28,29].

#### 3.2.2. FT-IR Analysis

**Figure 10:**
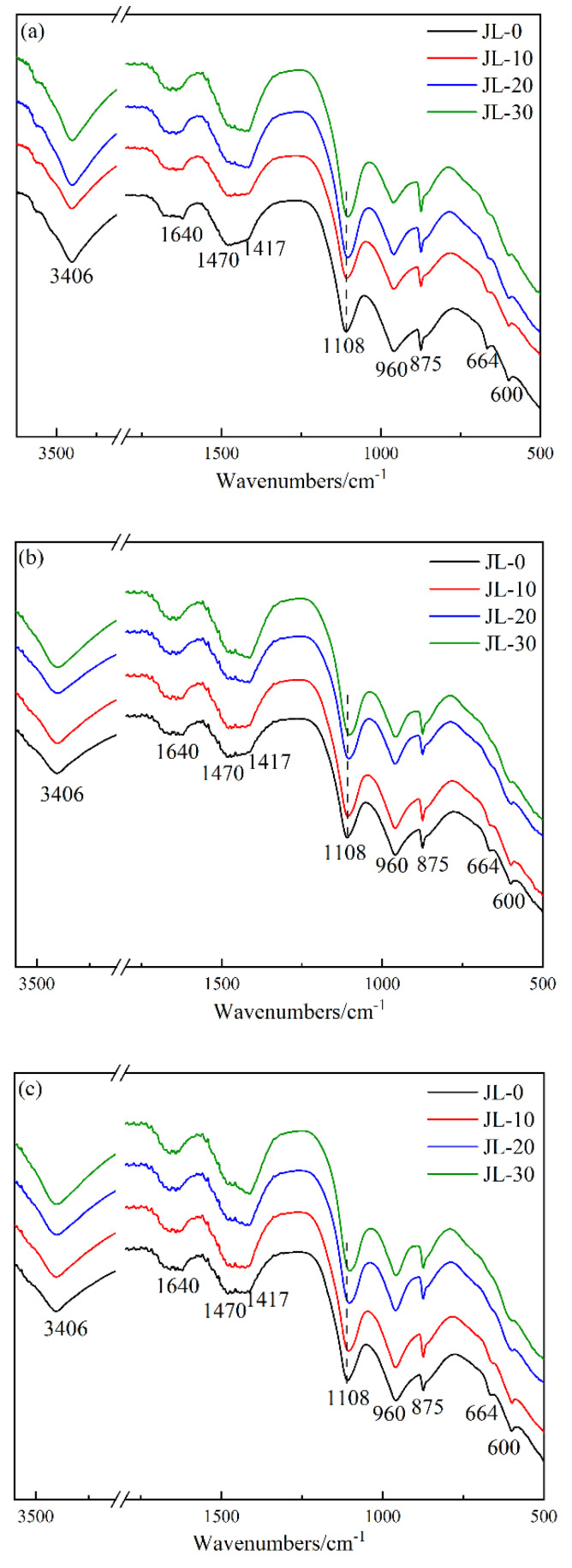
FT-IR spectra of hardened paste of solid waste-based cementitious material: (**a**) 3 d; (**b**) 7 d; (**c**) 28 d.

Figure 10 shows the infrared spectra of solid waste-based cementitious materials with different RS contents at 3, 7 and 28 days. It can be seen from Figure 10 that the infrared spectra are little affected by RS content or curing age. This indicates that RS content and curing age do not affect the type of hydration products, which is consistent with the results of the XRD analysis. The absorption peaks at 3406 cm^−1^ and 1640 cm^−1^ are attributed to the vibration of O-H bonds in structure water or crystalline water in the hydration products [30,31,32]. 1108 cm^−1^ is the vibration absorption peak of SO_4_^2−^. With the increase in RS content, the peak shifts to a lower wave number, and the peak shape becomes wider, indicating that the gypsum content decreases and more AFt is generated. The absorption peak at 960 cm^−1^ is attributed to the stretching vibration of Si-O bonds in the silica tetrahedron, and is the characteristic peak of C-(A)-S-H gels [30,33]. The peak at 600 cm^−1^ is a symmetric stretching vibration absorption band of Al-O-Si bonds, which mainly exist at the junction of Si-O tetrahedrons and Al-O tetrahedrons in GGBS [34]. The peak intensity decreases with RS content, indicating that RS can accelerate the dissociation of Si-O and Al-O tetrahedrons in GGBS, thus accelerating the hydration reaction rate of solid waste-based cementitious materials, which is not seen in the XRD analysis. The absorption peak at 1417 cm^−1^ is attributed to the characteristic absorption band of CO_3_^2−^ due to the samples’ erosion and carbonization by CO_2_ in the air during curing [31,35].

#### 3.2.3. SEM Analysis

**Figure 11:**
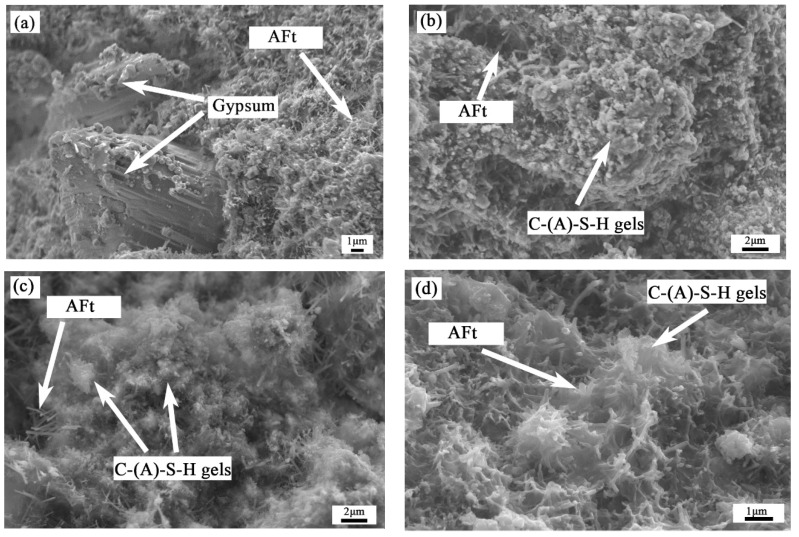
SEM micrographs of solid waste-based cementitious material: (**a**) JL-0-3d; (**b**) JL-0-28d; (**c**) JL-20-3d; (**d**) JL-20-28d.

Figure 11 shows the SEM morphologies of two hardened paste groups, JL-0 and JL-20, at 3 d and 28 d. In Figure 11a,d, there are a large number of needle/rod-like AFt crystals and amorphous C-(A)-S-H gels. Thus, there is no significant difference in the types of hydration products of JL-0 and JL-20. It can be seen from the comparison between 3-day and 28-day specimens that, with the extension of curing age, the size of AFt becomes larger, and the morphology of the gels varies from pellet and flocculent to shelf and layer. After curing for three days, the gels and AFt structures are relatively loose with more pores. When cured for 28 days, the gels and AFt show interwoven growth, and most of the AFt is wrapped by gels, forming a more compact structure. In addition, compared to the 3-day specimen, there are a few large holes (>1 μm) on the surface of the 28-day paste.

As shown in Figure 11a,b, the length of AFt in JL-0-3d is about 1–2 μm, whereas that in JL-20-3d is about 2–3 μm, showing that AFt is more numerous and more robust. In addition, the amount of gel in the JL-0-3d specimen is less, showing a dispersed spherical shape and failing to cover the AFt. However, the gels in the JL-20-3d specimen are flocculent, wrapping AFt more tightly. Unreacted gypsum is also found in Figure 11a and shows a short-thick cylindrical morphology. Based on XRD analysis, adding RS can promote the early-stage hydration of solid waste-based cementitious materials.

#### 3.2.4. TG-DSC Analysis

**Figure 12:**
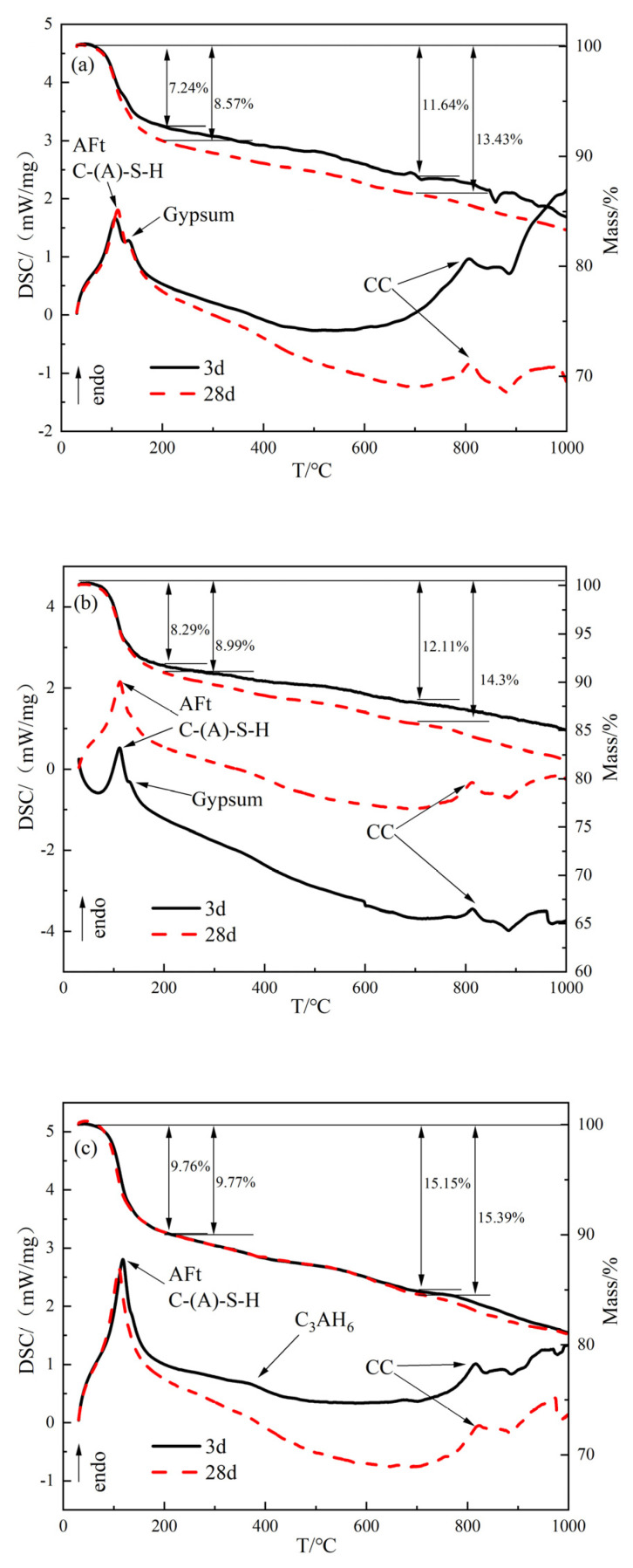
TG-DSC curves of the hardened paste of solid waste-based cementitious materials: (**a**) JL-0; (**b**) JL-10; (**c**) JL-20.

Figure 12 shows the TG-DSC curves of hardened paste of slurry specimens in JL-0, JL-10, and JL-20 samples after hydration for 3 d and 28 d. As can be seen from the DSC curves in Figure 12a, the obvious endothermic peak at 110 ℃ is attributed to the crystalline water removal from C-(A)-S-H gels and AFt [36,37]. The endothermic peak at 830 °C is attributed to the decomposition of CaCO_3_. The biggest difference between the DSC curves of the JL-0 sample under curing at 3 d and 28 d is the thermal decomposition peak of gypsum at 133 °C, which is more significant at 3 d but is not easily found at 28 d. The difference indicates that the gypsum content decreases significantly as the curing age extends, which is consistent with the XRD analysis results. It is also seen from Figure 12b,c that with the increasing RS content, the DSC curves undergo two variations:

(1) The area of the endothermic peak of gypsum gradually decreases in all samples and completely disappears in the JL-20 sample, indicating that the addition of RS increases the consumption of desulfurized gypsum;
(2) For the DSC curve of JL-20, there is a widened endothermic decomposition peak of C_3_AH_6_ at 364 °C after hydration for 3 d, indicating that the hydration of RS generates the intermediate product C_3_AH_6_. When the RS content is 20%, the generated C_3_AH_6_ is not entirely consumed. However, this endothermic decomposition peak is not found on the curve after hydration for 28 days, indicating that C_3_AH_6_ is wholly consumed at that time.

By observing the TG curves in Figure 12a, it can be found that the weight loss of JL-0 after hydration for three days and 28 days is 7.24% and 8.57%, respectively, when the temperature is lower than 200 °C. More weight loss indicates that hydration products are continuously generated during curing. However, the difference between the weight losses of the JL-10 and JL-20 samples is relatively small. This indicates that hydration products are mainly formed in the early stage after RS is added, and that the hydration rate significantly slows down after three days. At temperatures below 700 °C, weight loss results from the interlayer water and crystalline water being removed from hydration products (i.e., C-(A)-S-H gels and Aft). From the TG curves (Figure 12), the weight losses of JL-0, JL-10, and JL-20 samples before 700 °C are found to be 11.64%, 12.11%, and 15.15%, respectively, after three days of curing and 13.43%, 14.30%, and 15.39%, respectively, after 28 days of curing. The weight losses all increase with the content of RS, indicating that the amounts of C-(A)-S-H gels and AFt increase with RS addition.

#### 3.2.5. Heat of Hydration

**Figure 13:**
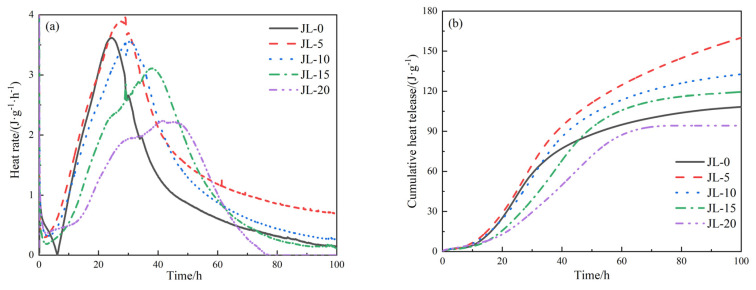
The hydration heat of solid waste-based cementitious material within 100 h: (**a**) heat release rate in hydration; (**b**) cumulative hydration heat release.

Figure 13 shows the hydration heat release curves of solid waste-based cementitious materials in cemented backfill over 100 h. It can be seen from Figure 13a that the exothermic hydration process of JL-0 is very similar to those of JL-5–JL-20. As seen in JL-0–JL-20 curves, the starting points of the acceleration periods are 6.25 h, 2.37 h, 2.57 h, 2.62 h, and 3.12 h, respectively. This indicates that RS addition shortens the dormant period of hydration and makes the acceleration stage happen earlier. The earlier the hydration process enters the acceleration period, the faster the hydration, which shortens the setting time of backfill and is consistent with the results in Figure 7.

Comparing the peaks consisting of the hydration acceleration and deceleration periods shown in Figure 13a, the widths of JL-15 and JL-20 are significantly larger than those of JL-0–JL-10, and the peak heights are reduced. This can be because when the RS content is higher, the GGBS content is lower. After the RS is rapidly hydrated, the substances that can be hydrated in the cementitious materials become less, thus reducing the hydration rate.

Based on Figure 13b, after mixing with water, the hydration heat release of the cementitious material increased gradually within 100 h. The hydration heat release of JL-0–JL-20 samples is 108.26 J·g^−1^, 160.06 J·g^−1^, 132.73 J·g^−1^, 119.46 J·g^−1^, and 94.17 J·g^−1^, respectively; the hydration heat release of JL-5 to JL-15 samples is higher than that of JL-0 while the hydration heat release of JL-20 is lower than that of JL-0. Therefore, when RS content is less than 15%, adding RS can increase the number of hydration products (i.e., C-(A)-S-H gels and AFt) and promote the hydration of solid waste-based cementitious materials. However, excessive RS content can negatively affect the hydration degree of solid waste-based cementitious materials.

## 4. Discussion

### 4.1. Hydration Mechanism of Solid Waste-Based RS-GGBS-SS-DG Quaternary Cementitious Materials

The hydration process of solid waste-based RS-GGBS-SS-DG quaternary cementitious materials can be mainly divided into three stages, i.e., (1) dissolution of raw materials, (2) formation of hydration products, and (3) cementation and condensation of hydration products.

(1) Dissolution of raw materials

Table 4 lists the different ionic groups formed in the liquid phase after the solid waste-based cementitious materials are mixed with water. As shown in Equation (5), C_12_A_7_ in the RS reacts quickly after contacting water, releasing a large number of aluminum-containing groups [38]. This increases the heat release during the hydration of the cementitious material and reduces the dormant period. DG can be spontaneously dissociated in an aqueous solution, as shown in Equation (6).

(5) $${12\mathrm{CaO}\cdot 7\mathrm{Al}}_{2}O_{3}{+51H}_{2}O\to {3(4\mathrm{CaO}\cdot \mathrm{Al}}_{2}O_{3}{\cdot 13H}_{2}O)+4\mathrm{Al}{\left(\mathrm{OH}\right)}_{3}$$

CaSO_4_·2H_2_O→Ca^2+^ + SO_4_^2−^ + 2H_2_O (6)

C_2_S, C_3_S, and C_3_A in SS can form C-S-H gels and Ca(OH)_2_ after reacting with water [39], as shown by Equations (7) and (8).

3CaO·SiO_2_ + H_2_O→Ca^2+^ + OH^−^ + (H_2_SiO_4_)^2−^ + (H_3_SiO_4_)^−^ (7)

3CaO·Al_2_O_3_ + H_2_O→Ca^2+^ + OH^−^ + (H_3_AlO_4_)^2−^ (8)

The dissolution of RS and SS both increase the pH of the liquid phase and form an alkaline environment that can promote GGBS dissolution. OH^−^ can crack the Si-O bonds and Al-O bonds in the glass phase of GGBS, releasing silicate ions (active SiO_2_) and aluminate ions (AlO_2_^−^) [40].

In the alkaline environment, the depolymerized Si-O and Al-O bonds in the GGBS undergo secondary bonding to generate Si-O tetrahedrons ([H_3_SiO_4_]^−^), Al-O tetrahedrons ([H_3_AlO_4_]^2−^), and Al-O octahedrons ([Al(OH)_6_]^3−^). The reaction equations are shown in Equations (9)–(11) [11,41,42].

(9) $${\mathrm{SiO}}_{2}{+\mathrm{OH}}^{-}{+H}_{2}O\to {\left[H_{3}{\mathrm{SiO}}_{4}\right]}^{-}$$

(10) $${\mathrm{AlO}}_{2}^{-}{+\mathrm{OH}}^{-}{+H}_{2}O\to {\left[H_{3}{\mathrm{AlO}}_{4}\right]}^{2-}$$

(11) $${\mathrm{AlO}}_{2}^{-}{+\mathrm{OH}}^{-}{+H}_{2}O\to {{\left[\mathrm{Al}(\mathrm{OH}\right)}_{6}]}^{3-}$$

(2) Formation of hydration products

The XRD, FT-IR, TG-DSC, and SEM results confirmed that the hydration products of the solid waste-based cementitious material system are mainly C-(A)-S-H gels and AFt, which do not vary significantly after RS addition. After the solid waste-based cementitious material is dissolved, [Al(OH)_6_]^3−^ combines with SO_4_^2−^ and Ca^2+^ in the liquid phase to generate AFt (Equation (12)) [41]. In addition, C_12_A_7_ in RS also combines with SO_4_^2−^ and Ca^2+^ dissolved from gypsum to generate AFt [13,19,43,44,45,46], as shown in Equation (13). The increase in RS content also increases the aluminate content in raw materials, thus accelerating the generation of hydration products, especially AFt.

(12) $${6\mathrm{Ca}}^{2+}{+\;3\mathrm{SO}}_{4}^{2-}{+\;2[\mathrm{Al}(\mathrm{OH})}_{6}{]}^{3-}{+\;26H}_{2}O\to 3\mathrm{CaO}\cdot {\mathrm{Al}}_{2}O_{3}\cdot {3\mathrm{CaSO}}_{4}\cdot {32H}_{2}O$$

(13) $${12\mathrm{CaO}\cdot 7\mathrm{Al}}_{2}O_{3}{+\;12\mathrm{CaSO}}_{4}{+\;137H}_{2}O\to {4(C}_{3}{A\cdot 3\mathrm{CaSO}}_{4}{\cdot 32H}_{2}O)+\;6\mathrm{Al}{\left(\mathrm{OH}\right)}_{3}$$

While [H_3_SiO_4_]^−^ can combine with Ca^2+^ to form C-S-H gels (Equation (14)), it can also react with Ca^2+^ and [H_3_AlO_4_]^2−^ together to form C-A-S-H gels (Equation (15)) at the same time [41].

(14) $${\left[H_{3}{\mathrm{SiO}}_{4}\right]}^{-}{+\mathrm{Ca}}^{2+}\to C-S-H$$

(15) $${\left[H_{3}{\mathrm{SiO}}_{4}\right]}^{-}+{\left[H_{3}{\mathrm{AlO}}_{4}\right]}^{2-}{+\mathrm{Ca}}^{2+}\to C-A-S-H$$

(3) Cementation and condensation of hydration products

Under the van der Waals force, C-(A)-S-H gels attach to the surface of unreacted material particles, cementing the dispersed particles in the slurry into a whole system so that the slurry has a certain initial compressive strength. Currently, the structure of the C-(A)-S-H gels is relatively loose, and the system is not compact. The gel’s pores are embedded in the needle/rod-shaped AFt, making the slurry structure dense. With the increase in curing age, the amount of C-(A)-S-H gels and AFt increases, and slurry density increases accordingly. Various hydration products are condensed and interwoven, forming an overall dense structure.

### 4.2. Effects of RS on Properties of Cemented Solid Waste-Based Backfill

Compared to cement backfill, solid waste-based cementitious backfill performances are poorer, including bleeding rate, setting time, fluidity, and early-age compressive strength. However, the performances can be enhanced by the RS addition. Within the RS content range of 0–40%, the bleeding rate of backfill slurry shows a declining trend, decreasing from 10% to 3% as RS content increases. The reduction in bleeding rate is conducive to enhancing the water retention performance of slurry. This trend is very beneficial for reducing the void caused by bleeding water during mine filling and improving filling efficiency. In addition, the fluidity of solid waste-based backfill slurry also increases with RS addition and reaches the same grade as that of cement.

The higher the RS amount, the faster the early-stage hydration rate of solid waste-based cementitious material, the shorter the dormant period of hydration, the higher the hydration heat, and thus the shorter the corresponding setting time of the backfill. In addition, with the increase in RS content, more AFt is generated, meaning more water is consumed, which indirectly increases the concentration of backfill slurry and significantly enhances the compressive strength of the backfill. When 10–30% RS is added, the compressive strength is 20% higher than the cement backfill, effectively overcoming the difficulty of demolding or operating due to inadequate early-age strength. However, when the RS amount is excessive, the amount of GGBS is insufficient, which leads to a reduction in the 28-day compressive strength of backfill. When the RS content is between 5% and 15%, the performance of solid waste-based backfill can be enhanced, and 15% RS leads to the most pronounced enhancement effect.

### 4.3. Cost Analysis of Solid Waste-Based Cementitious Material

**Figure 14:**
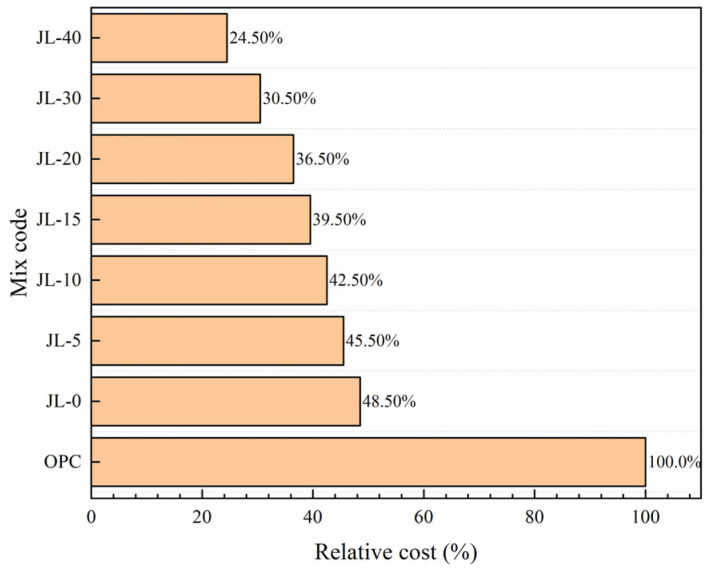
Comparison of relative cost between the quaternary solid waste-based cementitious materials and cement.

Compared to OPC, a prominent feature of solid waste-based quaternary cementitious material is that its main raw materials are all industrial solid wastes. Thus, it has significant advantages in reducing the cost of raw materials. The RS-GGBS-SS-DG quaternary solid waste-based cementitious material and cement cost are compared and analyzed without considering the influence of aggregate cost. The SS and GGBS used in this study are common concrete-mineral admixtures widely used to substitute cement to prepare building materials. Their prices are about 40% and 60% of the cement price, respectively. Further, DG, as a retarder, is often used to adjust the setting time of cement, and its price is about 10% of that of cement. In addition, RS is obtained as a kind of waste slag from steel mills. There is still no good way to use the RS in bulk, so the price is low or negligible. Figure 14 compares the costs of solid waste-based cementitious materials and cement under different RS content conditions. It indicates that, given the same amount of cementitious materials, the quaternary solid waste-based cementitious materials have great advantages over cement in terms of economic cost. In addition, the higher the content of RS, the more obvious the advantage. When the RS content increases by 5%, the economic cost relative to cement can be reduced by 3%. Although cement is usually mixed with GGBS or fly ash to prepare cemented backfill, the cement content is generally greater than 70%, and its cost is still much higher than that of RS-GGBS-SS-DG solid waste-based cementitious materials. Considering the properties of solid waste-based cemented backfill, when the RS content is 10–20%, the performance of solid waste-based cemented backfill is comparable to that of cement backfill, which can meet the requirements for general mine filling. Therefore, the cemented RS-GGBS-SS-DG solid waste-based backfill has better application prospects and economic benefits.

## 5. Conclusions

In this paper, a novel solid waste-based quaternary cementitious material for mine filling was prepared using RS, GGBS, SS, and DG as raw materials. The variation in the hydration of cementitious material and the backfill performances under different RS contents were emphatically investigated.

RS addition can significantly enhance the early-age hydration performances of solid waste-based cementitious materials. The main hydration products of the RS-GGBS-SS-DG quaternary cementitious material systems are C-(A)-S-H gels and AFt. In addition, the amount of AFt significantly increases with the increase in RS content. In the RS content range of 0–15%, the bleeding rate of backfill slurry decreases continuously with the increase in RS content, mainly because AFt is a water-rich mineral and combines a large amount of free water. A large amount of aluminate (C_12_A_7_) in the RS significantly increases the early-stage hydration rate, and the 3-day compressive strength of backfill is significantly enhanced. The 3-day and 28-day compressive strengths are better than cement when the RS content ranges from 10% to 20%.

In the RS-GGBS-SS-DG quaternary solid waste-based cementitious material, the OH^−^ generated by the SS and RS hydration can accelerate the depolymerization of the glassy phase in GGBS and provide a sufficiently alkaline environment for the formation of C-(A)-S-H gels. The increase in RS content reduces the dormant period of hydration and makes the hydration acceleration period start earlier, thus accelerating the overall hydration reaction of cementitious materials. After the GGBS is replaced by RS in equal amounts, when the RS content is 10–20%, solid waste-based cementitious materials have better performance and a lower economic cost than cement.

In general, adding RS can overcome the problems of insufficient early-age strength and long setting time of solid waste-based cementitious material and significantly enhance the backfill performance. The results presented in this paper provide a new direction, with scientific and powerful data supporting the bulk utilization of RS and the preparation of new solid waste-based mine-filling materials with good working performance and mechanical properties.

## Figures and Tables

**Table 1:** Chemical composition (oxides) of cementitious materials (wt.%).

Materials	CaO	SiO_2_	MgO	Fe_2_O_3_	Al_2_O_3_	MnO	SO_3_	TiO_2_	K_2_O	Na_2_O	P_2_O_5_
RS	49.05	10.7	5.09	9.92	18.20	3.11	1.51	-	0.08	0.16	-
SS	38.80	16.30	7.53	22.70	3.26	2.93	0.51	-	0.09	0.11	1.75
GGBS	40.90	29.20	8.18	0.51	15.20	1.13	1.92	1.55	0.46	0.41	-
DG	41.20	3.65	6.03	1.34	0.68	0.04	44.80	-	0.07	0.11	-

**Table 2:** Chemical composition of tailings sand (wt.%).

Composition	CaO	SiO_2_	MgO	Fe_2_O_3_	Al_2_O_3_	MnO	SO_3_	K_2_O	Na_2_O
Content	23.1	36.6	13.3	7.9	7.88	0.17	2.05	1.75	1.45

**Table 3:** Mix proportions of RS-GGBS-SS-DG cementitious material (%).

Mix Code	RS	GGBS	SS	DG	Cement
OPC	0	0	0	0	100
JL-0	0	65	20	15	0
JL-5	5	60	20	15	0
JL-10	10	55	20	15	0
JL-15	15	50	20	15	0
JL-20	20	45	20	15	0
JL-30	30	35	20	15	0
JL-40	40	25	20	15	0

Note: JL-0 sample indicates that the RS amount is 0%. Other samples are similarly named.

**Table 4:** Dissociation of RS-GGBS-SS-DG solid waste-based cementitious materials in liquid phase.

Raw Material	Pozzolanic Activity	Dissolved Products	Reaction Conditions
RS	low	Al^3+^,OH^−^,Ca^2+^	N/A
GGBS	high	active SiO_2_, AlO_2_^−^	basic environment (OH^−^)
SS *	low	Ca^2+^, OH^−^, M^2+^	N/A
DG	N/A	SO_4_^2−^, Ca^2+^	N/A

* M^2+^ in SS represents divalent metal oxides such as oxides of Mg^2+^ and Fe^2+^.

## Data Availability

The data presented in this study are available on request from the corresponding author. The data are not publicly available due to the privacy restrictions.

## Abbreviations

RS, refining slag; GGBS, ground granulated blast furnace slag; SS, steel slag; DG, desulfurized gypsum; AFt, ettring-ite; C-S-H gels, calcium silicate hydrate gels; C-A-S-H gels, calcium aluminate hydrate gels; OPC, ordinary Portland cement; RH, relative humidity; XRF, X-ray fluorescence analysis; XRD, X-ray diffraction analysis; FT-IR, Fourier transform infrared ray diffraction analysis; SEM, scanning electron microscope; TG-DSC, thermogravimetric analysis and differential scanning calorimetry.
